# Understanding tonic and phasic irritability in developmental psychopathology among help-seeking children and adolescents in Switzerland: Protocol for the longitudinal multimodal UTOPICA study

**DOI:** 10.1136/bmjopen-2025-111168

**Published:** 2026-04-22

**Authors:** Sebastien Urben, Julia Giovannini, Aline Dietsche, Marion Abi-Kheir, Leon Schmidt, Julian Koenig, Argyris Stringaris, Kerstin Jessica Plessen, Michael Kaess, Ines Mürner-Lavanchy

**Affiliations:** 1 Division of Child and Adolescent Psychiatry, Lausanne University Hospital and University of Lausanne, Lausanne, Switzerland; 2 Division of Youth Mental Health, University of Basel, Basel, Switzerland; 3 Department of Child and Adolescent Psychiatry, Psychosomatics and Psychotherapy, University Hospital Cologne, Cologne, Germany; 4 Child and Adolescent Psychiatry, University College of London, London, UK; 5 University Hospital of Child and Adolescent Psychiatry and Psychotherapy, University of Bern, Bern, Switzerland

**Keywords:** Child & adolescent psychiatry, Surveys and Questionnaires, Task Performance and Analysis, Adolescent

## Abstract

**Abstract:**

**Introduction:**

Irritability represents one of the most common causes of referral to child and adolescent mental health services. Conceptually, tonic irritability (i.e., persistent grumpy mood) can be distinguished from phasic irritability (i.e., temper outbursts). The objective of this research project is to develop a fine-grained, ecologically valid and multimodal characterisation of tonic and phasic irritability to better understand the differential role of the two components in developmental psychopathology.

**Methods and analysis:**

The study has a longitudinal observational and experimental design and involves two sites: (a) the Division of Child and Adolescent Psychiatry at the University Hospital of Lausanne and (b) the Division of Youth Mental Health at the Faculty of Psychology at the University of Basel. 220 help-seeking and healthy youths aged 8–14 years and their families will participate in the study consisting of a baseline assessment (i.e., self-report, interviews, cognitive assessments, autonomic measures, as well as in-situ experiments), an ecological momentary assessment (EMA) phase (over 2 weeks, including experience sampling method, cognitive assessment and passive monitoring) and a 1-year follow-up. Statistical analyses will include multilevel regression (e.g., linear mixed modelling).

**Ethics and dissemination.:**

We obtained ethical approval from the local ethics committees (Cantonal Research Ethics Commission on Human Beings, CER-VD, #2023-01846) and data collection began in January 2025. The results of the present study will be published in peer-reviewed scientific journals and will be presented at key conferences in the field of child and adolescent mental health, as well as at conferences focusing on EMA. Additionally, findings will be disseminated to practitioners, the educational sector and associations working with youths. We further intend to make the findings accessible to the general public through social media, for instance.

**STRENGTHS AND LIMITATIONS OF THIS STUDY**

- This study adopts a multimodal and multiple informants’ perspective.
- This study integrates a micro- (ecological momentary assessment over 14 days) and macro-longitudinal (1-year follow-up) design.
- Ecological momentary assessment allows for the investigation of within- and between-person variability in tonic and phasic irritability.
- Although this study includes physiological measures, it does not measure neuronal substrates of irritability.

## Introduction

### Irritability: a highly prevalent psychiatric symptom in childhood

 Irritability is defined as an increased proneness to anger in response to frustration compared with peers at the same developmental stage.^1 2^ For instance, children with pathological irritability have a lower threshold to express anger and therefore exhibit recurrent temper tantrums inappropriate to the situation.^3 4^ Irritability represents one of the most common causes of referral to child and adolescent mental health services.^5^,^7^ Prevalence estimates range from 20%–30% for a broad definition of irritability^5 8^ to 1%–3% for severe chronic forms of irritability.^9^,^12^ Pathological irritability is listed as a cardinal or associated symptom in many affective and behavioural disorders listed in the fith edition of the Diagnostic and Statistical Manual of Mental Disorders.^13^ Irritability is associated with long-term adverse outcomes, including low educational attainment, poor health, higher delinquency, suicidality and a higher risk for adult depression, anxiety and conduct problems.^12 8 14^,^22^ As such, irritability in childhood has been postulated to be a transdiagnostic marker of psychopathology crossing externalising and internalising dimensions.^2123^,^28^

### Two components of irritability

Previous studies mainly characterised irritability as a unitary clinical phenomenon, resulting in a poor definition of irritability and a lack of a clear understanding of its clinical correlates and pathophysiology. Most recent conceptualisations distinguish tonic from phasic irritability.^29^,^32^ While tonic irritability refers to a persistently angry, grumpy, bad-tempered or grouchy mood, usually lasting days and weeks, phasic irritability refers to behavioural outbursts of intense anger, manifested by brief or protracted verbal or physical aggression.^30 33 34^ The clinical distinction, as well as the boundaries between tonic and phasic irritability, is highly correlated; however, they are distinguishable^35 36^ already at 3 years of age.^37^ Further, recent studies showed that, in adolescence, the two components of irritability showed distinct concurrent correlates and early antecedents^38^ and distinct patterns of development and co-development, suggesting discriminant validity^39^ that may also be identified by parents and teachers.^40^

The distinction between phasic and tonic irritability has emerged in the context of investigations focused on disruptive mood dysregulation disorder (DMDD), a diagnostic category recently introduced in psychiatric diagnostic manuals and characterised by the presence of both components.^31 41^

### Clinical correlates of tonic and phasic irritability

While tonic irritability seems to be more closely related to internalising psychopathology, phasic irritability has stronger links to externalising psychopathology.^35^ Indeed, in cross-sectional studies of help-seeking children between the ages of 5 and 18, phasic irritability was associated with attention-deficit/hyperactivity disorder (ADHD) symptoms^42^ and externalising problems but not internalising symptoms.^43^ Longitudinal studies in community children reported that tonic irritability predicted subsequent depression and generalised anxiety disorder, while phasic irritability was slightly more stable, rather heritable and more frequently predicted later ADHD, oppositional defiant disorder, conduct disorder and substance use disorder.^35 44^ The distinction between tonic and phasic irritability even allows for the distinction between symptom constellations in depression and suicidality.^45 46^ In another longitudinal study of a community sample of 13–22year-olds, tonic irritability predicted ADHD at age 16 and major depressive disorders at age 22, whereas phasic irritability predicted phobias and hypo-/mania at age 16.^29^ Nevertheless, in a longitudinal study of a community sample of 9–13-year-olds, tonic and phasic irritability predicted similar outcomes, while phasic irritability was more prevalent than tonic irritability.^33^ Regarding the mixed results observed in previous studies, further research is needed.

### Dynamics of irritability at the within-person level

Although irritability is omnipresent in distinct psychiatric disorders, its manifestation is highly heterogeneous across and within individuals. The reason for this might—at least in part—lie in its two components, which differentiate in their temporal dynamics. While tonic irritability persists for days, phasic irritability typically lasts for minutes to a couple of hours. The important question of whether tonic and phasic irritability are independent events or whether mood between tantrums is a series of less severe outbursts^34 47^ could be approached by using ecological momentary assessment (EMA). EMA includes the so-called experience sampling method (ESM), which consists of frequent, repeated self-report assessment of thoughts, cognition, experiences and behaviours^48 49^ via a handheld device—usually a smartphone.^50^ Moreover, EMA includes passive monitoring (e.g., actigraphy, monitoring of smartphone use), physiological measures, as well as cognitive EMA.

EMA has high ecological validity compared with traditional clinical diagnostics. Patient’s reports do not suffer from recall bias due to long-term retrospective assessment, which may be advantageous, as studies suggest that irritable youth may under-report levels of symptoms, retrospectively, in comparison to their peers.^24 51^ To date, irritability has been examined using parent or clinician ratings to examine irritability without distinguishing tonic and phasic dimensions, mainly at the between-person level. One notable exception is a very recent transdiagnostic study using ESM to examine pathological irritability and demonstrated the feasibility and reliability of measuring this symptom in 109 children and adolescents aged between 8 and 18 years.^52^ The authors observed convergent validity of ESM measures of irritability with clinical self-report and interview measures, as well as with a task inducing mild levels of frustration,^53 54^ namely the Stop Signal task (SST).^55^ Even though the authors assessed both components of irritability, they did not distinguish between the two components in their analyses. Thus, further studies are needed to refine the tonic-phasic distinction using EMA, including not only experience sampling but other modalities (e.g., cognitive or autonomic function).

### Time-course approach to cognitive and autonomic underpinnings of irritability

The cognitive and neurobiological pathways leading to pathological irritability have been hypothesised to be grounded in deficits in instrumental learning, which enable children to adapt their behaviour to respond most appropriately to environmental stimuli.^2^ In typical development, emotion regulation skills develop in response to frustration during the preschool period.^56 57^ While anger is a normative response to frustrative non-reward, the chronic negative affective response to non-reward might stem from a deficit in emotion regulation and eventually lead to pathological irritability. A key construct of emotion regulation is cognitive control (or inhibitory control), which has been posited as a crucial mechanism that may affect the generation and maintenance of irritability^2 58^ but has not been extensively investigated in this context yet.

Children with conduct disorders, autism and ADHD showed higher emotional responses alongside a lower autonomic regulation pattern (i.e., higher sympathetic arousal) in response to frustration.^659^,^62^ However, autonomic markers of emotion dysregulation have not been extensively studied in the context of irritability yet.^63 64^ The pattern of respiratory sinus arrhythmia (RSA: influence of respiration on heart rate) changes in response to frustration induction was related to better emotion regulation and adjustment (i.e., more prosocial behaviours) in community adolescents.^65^ Moreover, the duration of RSA recovery after emotion induction was found to be crucial in the distinction between functional and dysfunctional emotion regulation,^66 67^ highlighting the importance of assessing detailed time-courses of patients’ emotion reactivity in challenging situations.^68 69^ Such time courses can be measured using the ‘in-situ’ approach, which might reveal non-linear associations of dysfunctional psychophysiological processes in irritability.^70^ Preliminary findings showed that irritability is related to increased heart rate and decreased heart rate variability (HRV) during the performance of an SST, inducing mild frustration, in a transdiagnostic sample (n=51) presenting with irritability (i.e., ADHD, DMDD, anxiety disorders vs healthy controls).^53 54 71^ ‘In-situ’ studies inducing higher levels of frustration in children with irritability are, thus, warranted.

### Psychosocial environment

Further, the psychosocial environment and, in particular, the interpersonal dynamics of caregiver-child interactions may be of importance to understand irritability.^72^ Indeed, phasic irritability was uniquely related to self-reported maladaptive parenting.^36^ During development, parent–child interactions evolve to meet the needs of the child, resulting in many adjustments for both the parent (i.e., co-regulating their child’s emotions^73^) and the growing child,^74^ including irritability.^75 76^ Thus, the quality of parenting is important for the child’s self-regulation,^73 77^ as are the parent’s own self-regulatory resources to help the child develop adaptive anger management skills.

### Research gaps

A central question is whether tonic and phasic irritability reflect different intensity levels along a single continuum or constitute related but distinct phenotypes.^34^ This study uses ecologically valid, multimodal and multi-informant assessments, as well as EMA, to characterise both dimensions. By integrating cognitive, psychosocial, psychophysiological and autonomic measures, the study aims to clarify within-person dynamics and identify proximal markers of tonic and phasic irritability. This approach will also allow for the examination of their concurrent and longitudinal associations with clinical outcomes, with the goal of laying a foundation for future work improving early identification and intervention targets.

### The current research project

The overall objective of this research project is to develop a fine-grained, ecologically valid and multimodal characterisation of tonic and phasic irritability in order to better understand the differential role of the two components of irritability in developmental psychopathology. In particular, through ESM assessment, our *first aim* is to validate the tonic—phasic distinction of irritability at the within-person level and to determine the temporal dynamics as well as the natural context of expression of the two components. We hypothesise that the tonic and phasic components of irritability measured with ESM will be strongly and consistently associated with their respective well-established clinical measures, thereby providing new, reliable and complementary measures. We further assume that high levels of tonic irritability will precede phasic irritability (i.e., temper tantrum). Moreover, contextual information (including parenting) about the current psychosocial environment might be differently related to tonic vs phasic irritability.

Our *second aim* is to examine whether distinct cognitive and autonomic correlates of tonic and phasic irritability exist, both at the within-person level and at the between-person level. Related to this aim, we hypothesise that experimental measures of ambulatory cognitive control will be more strongly associated with phasic than tonic irritability. From the ‘in-situ’ assessment, we expect that HRV reactivity will be more specifically related to phasic aspects of irritability (clinical and ESM assessments), whereas HRV recovery (more related to emotion regulation) will be associated with tonic irritability (ESM and well-established clinical assessments). Finally, by means of the autonomic EMA, we expect to identify autonomic dysregulation concurrent with tonic irritability but probably preceding episodes of phasic irritability.

Finally, extending previous studies examining the distinction between tonic and phasic irritability in clinical samples, using our ecologically valid multimodal approach, our *third aim* is to use the fine-grained understanding of irritability to examine the unique and different associations of its tonic and phasic facets with current and future psychopathology, both at the within- and between-person level. We hypothesise that tonic irritability will be independently related to current and future internalising psychopathology, including depressive, anxiety symptoms and suicidal ideation, while phasic irritability can be independently associated with current and future externalising symptoms, hyperactivity symptoms, oppositional, conduct problems and impulsive suicide attempts. We will further explore whether EMA measures and ‘in-situ’ assessment are more strongly associated with current and future psychopathologies compared with well-established clinical assessments. Finally, we expect more stability in the phasic compared with the tonic component of irritability over the long term.

## Methods and analysis

This research project employs a longitudinal design combining observational and experimental elements. The project is a multi-centre study including two sites: (a) the Division of Child and Adolescent Psychiatry at the University Hospital of Lausanne and (b) the Division of Youth Mental Health at the Faculty of Psychology at the University of Basel. The data collection phase begins in January 2025 and will last until the end of 2027.

### Participants

We will recruit n=180 youths who are currently seeking help from various child and adolescent mental health services (as well as those who have sought help in the past 12 months). Moreover, we will recruit n=40 healthy children (i.e., not seeking help and no diagnosed psychiatric disorder). In addition, we will recruit participants’ parents (n=220–440). Thus, the total sample of the study will range from 440 to 660. Table 1 describes the inclusion and exclusion criteria. The sample size of a total of n=220 children was justified by a power calculation (see power calculation section).

**Table 1:** Inclusion/exclusion criteria for children and their legal guardians

Criteria	Children	Legal guardian
Inclusion		
Being aged 8–14 years	x	
Having sufficient intellectual abilities and sufficient language skills (German or French) to be able to participate in the study	x	x
Drug naïve (no psychotropic medication, no medication impacting heart rate)*	x	
Written informed assent or written informed consent	x	x
Exclusion		
Known cardiovascular disorders impacting the heart functioning, severe health conditions that currently demand intensive medical treatment (i.e., cancer, neurological disease, severe conditions related to mental health)	x	
Having a pacemaker	x	
Known diagnoses in schizophrenia or psychosis spectrum as well as in autism spectrum disorders at study inclusion	x	
Known active life crisis (i.e., intense suicidal ideas or behaviours)	x	

* As we aim to characterise the associations between these phenomena without the confounding influence of medication that may alter emotional, behavioural and psychophysiological functioning.

### Procedure

A standardised data-collection protocol was implemented to ensure methodological consistency across study sites. All assessment instruments have demonstrated strong psychometric properties and are validated in prior research.

**Figure 1:**
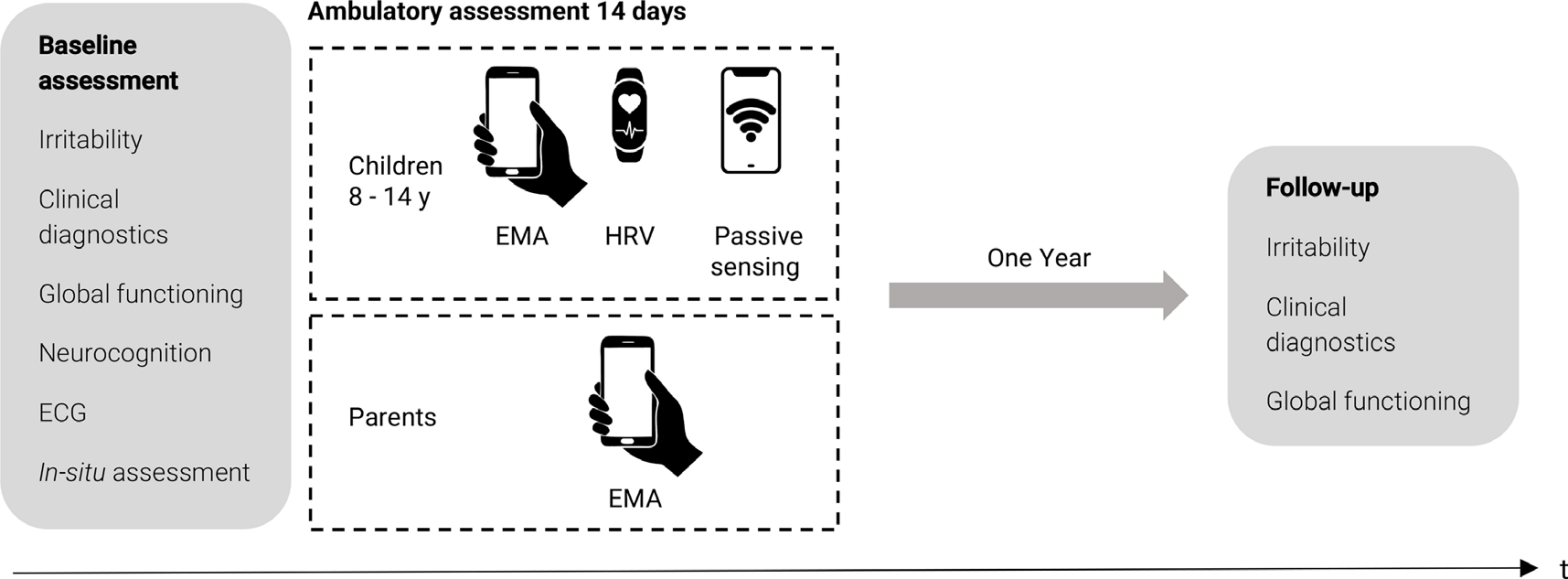
Study overview. Ecological momentary assessment (EMA) phase follows baseline assessment directly and includes ESM self-report and parent-report, as well as cognitive control task (hand holding smartphone symbol), ambulatory autonomic function using a wearable (wearable symbol) as well as passive smartphone sensing (smartphone wifi symbol). ‘In-situ’: situation mimicking daily life situation generating frustration. EMA, ecological momentary assessment; ESM, experience sampling method; HRV, heart rate variability.ECG:electrocardiogram

The project entails two main study phases: baseline and follow-up (see figure 1). During baseline, participants will undergo an extensive clinical assessment including interviews, self- and parent-ratings, self-control and intelligence testing, and electrocardiogram (ECG). Further, they will participate in an ‘in-situ’ assessment. In this part, the participant will be in a situation eliciting frustration (mimicking irritability) while various emotional, cognitive and psychophysiological parameters (i.e., ECG) will be examined. The clinical and ‘in-situ’ assessments will be conducted in one appointment. Participants will then conduct 2 weeks of EMA, consisting of ESM, an ambulatory cognitive task, ambulatory heart rate measurement and actigraphy, as well as passive smartphone sensing (for those using their own smartphone). Finally, participants and their caregivers will be reassessed at a 1-year follow-up. Given the intensive, multimodal nature of our protocol, a 1-year interval offers the optimal balance between capturing meaningful developmental changes in irritability-related psychopathology and maintaining feasibility, retention and ethical acceptability, consistent with current youth mental-health and early-risk-prediction studies. The 1-year follow-up assessment will include a clinical re-assessment of tonic and phasic irritability (see Measures section for details), general psychopathology, severity of psychopathology, global functioning, significant life events and treatment or help-seeking behaviours. Financial compensation will be provided in the form of vouchers to avoid direct monetary compensation that might be used inappropriately (e.g., cigarettes, alcohol or illegal substances).

### Measures

#### Baseline assessment

Interview and questionnaire data are collected via REDCap (Research Electronic Data Capture). Self-report measures are completed directly by the participants themselves either at home (before the first lab assessment) or at the lab during the assessment. For children aged 8–10 years, questionnaires can be completed with the support of a parent or the research team.

Demographic variables include age, sex, gender, pubertal stage, ethnicity, school form, chronic illnesses, medication intake, visual acuity and therapeutic follow-up. Psychosocial variables include socioeconomic status, family composition and marriage status of parents. Physical activity, sleep, traumatic events and screen use will also be assessed.

Psychiatric diagnoses will be obtained using the clinical interview Kiddie Schedule for Affective Disorders and Schizophrenia.^78^ Severity of psychopathology (Clinical Global Impression Scale^79^ and global functioning Children’s Global Assessment Scale^80^) will be assessed by the interviewer.

Irritability will be assessed comprehensively using the Affective Reactivity Index^81^; the Child Behavior Check List (for parents and young children—8–10 years old)/Youth Self-Report (for youths from 11 to 14 years old)^82^; and a questionnaire specifically developed to measure phasic and tonic components of irritability (the Tonic and Phasic Irritability Scale).^32^

The short version of the Difficulties in Emotion Regulation Scale^83^ and the Diagnostic Tool for Affective Dysregulation in Children–Screening Questionnaire^84^ will be used to assess emotion regulation problems (also rated by the parent). The Brief Self-Control Scale^85^ will quantify general trait self-control. The Anger Rumination Scale^86^ will assess the rumination (i.e., unwanted thoughts, emotions) related to past events eliciting anger. Regarding sleep quality, chronotype will be assessed with the Morningness-Eveningness Scale for Children^87^ and daytime sleepiness with the Epworth sleepiness scale adapted for children and adolescents.^88^ Adverse childhood events will be measured with the Adverse Childhood Experiences scale.^89^ Detailed information about routine clinical care will additionally be collected during the whole study phase.

The Symptom Checklist-90-R^90^ will measure psychopathological symptoms of parents. The Brief Irritability Test^91^ will measure parents’ irritability and the Tonic and Phasic Irritability scale^32^ will measure children’s irritability. The short version (20 items) of the Parental Affect Test^92^ will evaluate how parents deal with negative situations with their children. The short version of Being a Parent Scale^93^ will assess measures of parenting satisfaction and efficacy. The short version (28 items) of the Parenting Styles and Dimensions Questionnaire^94^ will measure the three parenting styles, namely authoritative, authoritarian and permissive parenting. The Parent-Child Coercive Process Scale^95^ measures coercive processes in parent-child dyads.

General intelligence will be assessed with the five subtests Vocabulary, Block Design, Figure Weights, Digit Span and Coding of the Wechsler Intelligence Scale for Children-Fifth Edition^96^ as this short version has been demonstrated to provide an accurate estimation of general intelligence.^97^

*Cognitive control* will be assessed with an SST.^98 99^ The SST will consist of performing a choice reaction while restraining from responding when facing a stop signal. Patients must decide if the face presented is male or female by pressing one of two response keys. Stimuli consist of neutral and angry facial expressions from 12 individuals (6 females and 6 males; Macbrain Face Stimulus Set, www.macbrain.org). The stop signal consists of a red square appearing around the faces after a delay (initially set at 250 ms) that is adjusted depending on the participant’s success in inhibiting responses in the preceding stop trials. In cases of successful response inhibition, delay increases, and in cases of failed response, delay decreases for the next stop trial. This adjustment is supposed to result in a mean success rate for stop trials of about 50%, allowing for a good assessment of the stop-signal reaction time (SSRT).^100^ Presentation will be randomised across participants to avoid practice effects due to the repetition of the presentation of the stimuli. Three main scores will be computed: SSRT (time during which the inhibition of an already initiated response is possible), proactive adjustments (slowed: cautious; speeded: risky) and reactive adjustments (error-monitoring). An analogous task (i.e., go/no-go) will repeatedly be used during the EMA phase (see Ecological momentary assessment section).

During the *delay discounting* task, children choose between a varying amount of hypothetical money now or a hypothetical 100 CHF after a varying delay. The immediate money varies from 0 to 100 CHF in 5 CHF increments. The delayed money (always 100 CHF) is available after one of four delays (now, in 7, 30 or 365 days). Choices will be randomly displayed on the right and left sides of the screen. Three additional questions will be used to assess attention to the task (100 CHF in X days vs 100, 95 and 90 CHF now). For each of the delays, a double-limit algorithm adjusts the immediate reward based on previous choices in order to narrow the range of values. It then converges into a so-called indifference value, which the subject considers to be equivalent to the delayed reward.^101^ Reward is typically discounted in a hyperbolic function that depends on the amount, delay and a free impulsiveness indicator *k*. This variable *k* is the main dependent task variable, which can be obtained by fitting a hyperbolic function to the indifference values for every delay. Larger *k* values imply greater reward devaluation. The task has been found to be feasible and valid in 7–9-year-old children.^102^

#### Psychophysiological measures

HRV will be measured during a 5 min baseline while patients complete a minimally demanding Color Detection Task (CDT).^103^ ECG will be recorded using an EcgMove4 sensor (movisens GmbH, Karlsruhe, Germany) attached to the participants’ chest at the base of the sternum using a flexible belt with two integrated electrodes. ECG signals will be recorded at a sampling rate of 1024 Hz. Reporting of HRV processing and analyses will adhere to the Guidelines for Reporting Articles on Psychiatry and HRV.^104^ After visual inspection, data will be processed in Kubios HRV 3.0 Premium, including manual correction of R-peak detection and artefact removal. The square root of the mean squared difference of successive inter-beat-intervals (RMSSD, in ms) will be calculated for each patient as a time-domain measure of vagal activity.^105^

#### In-situ assessment

The ‘In-situ’ assessment of frustrative non-reward will be conducted using a rigged Affective Posner Task.^62106^,^109^ Participants are asked to identify a target following a cue by button press (left or right). The task consists of three conditions differing in the feedback participants receive. In the first condition, participants receive feedback regarding the accuracy of their performance on each trial. In the second condition, participants win or lose monetary incentives based on their performance. In the third condition, feedback is manipulated: participants receive rigged feedback in about 60% of trials and accurate feedback in about 40% of the correct trials. On trials with rigged feedback, participants are informed that they were ‘too slow’ and lose money regardless of their actual reaction time. In all three conditions, auditory and visual feedback are provided depending on the participant’s response. Delta of accuracy between blocks will index impairment of cognitive control due to affective context. Self-perceived frustration tolerance will be measured at the end of the task.^110^ Subjective feelings throughout the task will be measured with items from the brief Positive and Negative Affect Schedule (PANAS).^111 112^ ECG measurement will be continued during the ‘in-situ’ assessment. At the end of the task, a recovery phase is introduced through the administration of the CDT cited earlier. Self-perceived frustration tolerance will be measured during the task with a short version (i.e., irritated, nervous, attentive, agitated, angry) of the PANAS.^111 112^

#### Ecological momentary assessment

The EMA includes four types of measures: ESM (i.e., self-report), ambulatory cognitive assessment, autonomic regulation as well as passive monitoring.

ESM will be prompted four times a day (morning, noon, afternoon and evening). Reminders will be sent two times for each prompt after 30 and 60 min. Those children who do not own a smartphone will respond to ESM on their parents’ smartphone. All participants will respond during 14 consecutive days (during school period, not holidays). The ESM encompasses items measuring context, location, sleep, affect (current and since the last prompt), aggression, violent ideations, anger rumination, self-control, tonic as well as phasic irritability and conflicts. Parents will answer questions regarding their children but also their own affect, self-control and interaction with their child (current and since the last prompt).

An event-related questionnaire will be sent each morning to parents in case they would like to individually report a child’s temper tantrum during any given moment of the day. This questionnaire can be filled out multiple times during the day.

To collect the cognitive ambulatory assessment, participants will perform the online go/no-go task (in line with the lab version^113^ two times a day (coupled with the morning and afternoon assessments), provided through Inquisit 6 (a software for administering psychological tests that gives the option of running tests online) and already used successfully in a previous study.^114^

Ambulatory autonomic regulation will be assessed using a wristband (i.e., Garmin vívosmart 5), during the whole EMA period (14 days). Inter-beat intervals (time between two successive pulse pressure waves) are obtained using PPG (photoplethysmography). Each heartbeat will be detected individually; furthermore, each beat will be accompanied by a binary confidence metric (high=1, low=0) indicating the measurement’s quality. PPG data will be preprocessed using Firstbeat analysis software to calculate RMSSD in 1-minute intervals. The Labfront and Garmin Connect apps will be installed on the youth’s or the parent’s smartphone during the baseline assessment, to enable data extraction at the end of the EMA period. Moreover, the Ethica Avicenna application (also installed on the participants’ smartphone) will record screen state, Bluetooth and app usage (only on Android). To approximate app usage in participants owning an iPhone, we will additionally prompt those participants to send screenshots of their screen time app covering the 2 weeks of the EMA.

To maximise EMA response compliance, we will monitor daily REDCap entries. Additionally, every 2 or 3 days, we will review the synchronisation status for both mobile applications (i.e., Labfront, Garmin Connect). Participants will receive an SMS reminder on their smartphone if a decline in compliance is detected. If compliance does not improve, a member of the research team will contact the participant by phone to identify potential difficulties, offer support and encourage re-engagement during this phase.

#### Follow-up assessment

The follow-up assessment will take place 1 year after the baseline assessment. The clinical assessment will be similar to the baseline and contain the same measures of tonic and phasic irritability, psychopathology, severity of psychopathology and global functioning. In addition, we will monitor whether participants received any form of therapy or no therapy at all. All significant life events that potentially happened between assessments will also be queried. Table 2 summarises all measures.

**Table 2:** Overview of the assessments

Assessment	Baseline		Ecological momentary assessment		Follow-up
Mode	Lab (+ remote)		Remote, 2–4 times a day		Lab
Participant (parent/child/both)	Child	Parent	Child	Parent	Both
Duration	3–3.5 hours	2.5–3 hours	3–6 min	3 min	2 hours
Instruments					
Sociodemographics	+	+			
Psychiatric diagnoses	+	+			+
Severity of psychopathology and global functioning	+				+
Emotional and behavioural problems	+	+			+
Emotion regulation	+				
Tonic and phasic irritability	+	+			+
Anger rumination	+	+			
Self-control	+	+			
Sleep	+				
Parental dimensions		+			
General intelligence	+				
Cognitive control	+				
In situ frustration (including ECG)	+				
Experience sampling			+	+	
Ambulatory cognitive control			+		
Ambulatory HRV			+		
Passive sensing			+		

ECG, Electrocardiogram; HRV, heart rate variability.

### Statistical analysis plan

We will assess cross-language measurement invariance (French vs German) for multi-item scales using multi-group confirmatory factor analysis, testing configural, metric and scalar (threshold) invariance based on model fit and Δcomparative fit index (CFI)/Δroot mean square error of approximation (RMSEA). If inter-site differences are detected, the site will be included as a covariate in the statistical models to control for potential confounding.

#### Aim 1

First, we will compute separate standardised composite scores for tonic and phasic irritability at the within- (from the ESM measures) and between-person level (from the interviews and questionnaires). To assess convergent validity, multilevel regression models will then be used to test for associations between the tonic irritability composite score from ESM measures and the tonic irritability composite score from the clinical measures. The same models will be computed for phasic irritability. To test our second hypothesis, we will compute a lagged logistic multilevel regression model where tonic irritability from one ESM sampling will predict the presence of phasic irritability (categorical score) in the next ESM sampling (within-person level). Finally, to investigate the role of the psychosocial context (including parenting), multilevel regression analyses will be used to assess the role of conflict and psychosocial context on tonic/phasic irritability at the within-person level.

#### Aim 2

To test the pathophysiological correlates of tonic and phasic irritability, we will compute a multivariate linear mixed model,^115^ which represents a statistical framework for analysing fixed and random effects on multiple outcomes (i.e., tonic vs phasic irritability).^116^ This approach enables us to compare the effects of the pathophysiological variables on tonic versus phasic irritability, which are two separate but correlated outcomes. The model will hence allow computing effect sizes that will be compared with assessing the common and unique pathophysiological correlates (i.e., ambulatory cognitive control, ‘in-situ’ HRV) of tonic and phasic irritability both at a within- and between-person level. Lagged multilevel regression models will further be used to assess the temporal dynamics of the pathophysiological (e.g., ambulatory HRV, actigraphy and cognitive control) correlates of tonic and phasic irritability at the within-person level.

#### Aim 3

Finally, to understand the role of tonic and phasic irritability in psychopathology, two approaches will be used. First, we will adopt a dimensional perspective and use the severity of psychopathology of internalising and externalising problems (as the outcome variables at the between-person level). We, thus, must reduce the ESM measures to single measures for each participant. To do so, we will compute the mean and RMSSD that will be used in hierarchical regression analyses, also including the measure of irritability from clinical (or baseline) assessment. As both dimensions of irritability will be correlated and thus share a great part of common variance, we will compute the unstandardised residuals from regression analyses (tonic irritability regressed on phasic irritability and conversely).^117^ These scores will be used in the following regression models in order to calculate the independent importance of one dimension of irritability in understanding the severity of psychopathology. Second, we will adopt a transdiagnostic approach and use multilevel regression analyses (i.e., means-as-outcomes models)^118^ as proposed in the only previous paper combining within- and between-person approaches^51^ to assess the differences in ambulatory tonic and phasic irritability (within-person level) across diagnoses (between-person; e.g., anxiety, depression, DMDD, ADHD and oppositional defiant disorder).

#### Exploratory analyses

Moreover, complementary analyses may be conducted to further understand the clinical value of tonic and phasic irritability. Regarding the cardinal role of irritability in psychopathology, network analyses investigating the interrelationships between irritability, psychopathological symptoms, cognitive control, the psychophysiological and neurobiological measures will be conducted to assess the temporal networks (i.e., within-person, time-series), contemporaneous networks (i.e., within-person, cross-sectional) and between-person networks (i.e., between-person, cross-sectional). Such analyses will provide important information on the temporal and reciprocal relationships among the processes.^119 120^ This may help to understand the centrality of irritability as well as the reciprocal dynamics between tonic and phasic irritability and their interplay with concomitant processes.^119^ In addition, we will conduct fragmentation analyses, which may provide additional information on stability or fluctuations of tonic irritability at the within-person level, compared with standard approaches of variability.^121^ These analyses will help us to determine a range of ‘normal’ variability and thus identify the events outside this range (i.e., between-state transition probabilities). This approach might help to understand the stages where tonic irritability becomes problematic and examine the psychophysiological correlates as well as the future outcomes (e.g., subsequent phasic episode, later conflict). Finally, measures from passive sensing collected in those participants who own a smartphone will be used to relate tonic and phasic irritability as well as its cognitive and autonomic correlates to changes in habits and behaviour (e.g., listening to music, physical activity, sleep, duration or frequency of smartphone usage). As data are obtained passively without interrupting participants and without their own reporting, this data is of highest ecological validity and may pave the way to advance the assessment of irritability in the future. The problem of multiple testing will be considered with appropriate statistical methods and interpretation to avoid type I error (false positives). All analyses will be performed using the R environment for statistical computing V.4.1.0.^122^

The statistical analyses described above were planned when this study was conceptualised. If, during the course of the study, circumstances arise that make this analysis inappropriate or if, in the meantime, better methods of analysis are identified, a different analysis may be performed and will be described in the study report and publication.

#### Power analyses

We approximated the power of the main analyses (i.e., the multilevel regression) by using Monte-Carlo simulation (n=1000 repetitions). Based on pilot studies (unpublished data), we assumed a correlation coefficient of r=0.75 between repeated measures, participant compliance of 82% and an additional dropout of 20%. For the concurrent model, 56 repeated measures for each participant (4× measure a day for 14 days) will be collected for n=220 participants. For the temporal model (or lagged model, predicting next measures), 42 measures (three lagged variables per day for 14 days) will be available for the 220 participants. We expect a correlation between tonic and phasic irritability of r=0.60.^35^ The approximated power of these analyses focuses on the effects of β_n_ (between-person level) and γ_n_ (within-person level) for the repeated measures. These models have a power of 0.99 to detect small effect sizes at the within-person level and a power of 0.94 to detect medium effect sizes at the between-person level. For the temporal model (lagged model), we will achieve a power of 0.91 to detect small effect sizes at the within-person level and a power of 0.82 to detect medium effect sizes at the between-person level.

#### Missing data

Missing data will be minimised through electronic data capture (via REDCap with built-in range checks and completion indicators). Patterns of missingness will be assessed. If data are missing at random, multiple imputation will be applied. If data are missing completely at random, analyses may additionally be performed using expectation-maximisation procedures. Results from analyses on the imputed dataset will be compared with complete-case analyses as part of a sensitivity analysis.

### Patient and public involvement

In this research project, youths were invited to provide feedback in the phase of project development and implementation. For instance, after discussion with a panel of youths, we incorporated gamification elements into the ESM, making the data collection process more interactive and enjoyable and enhancing participant engagement and adherence. As such, we conducted an intensive pilot phase to test the comprehensiveness of the questionnaires and the burden that might be placed on participants by the assessment procedures. A panel of youths was involved to provide feedback to the baseline assessment and the EMA phase. Their valuable feedback was used to refine the questions, ensuring they are clear, concise and easy to understand for all participants. Finally, we intend to involve patient and public involvement partners in the dissemination of the results to the general public.

### Ethics and dissemination

#### Ethics

This research project is conducted in accordance with the protocol, the Declaration of Helsinki, the principles of Good Clinical Practice, the Human Research Act and the Human Research Ordinance, as well as other locally relevant regulations. We have obtained ethical approval from the local ethical committee (Cantonal Research Ethics Commission on Human Beings, CER-VD, #2023-01846). After having received written information about the study, parents/guardians will give written informed consent for the participation of their child. The child will also give oral consent to participate in the study.

#### Ethical considerations

The main risk associated with filling out the questionnaires or tasks is that it may induce negative or uncomfortable thoughts or emotions. We will conduct a debriefing at the end of the assessments to discuss such issues and, if needed, refer the families to a mental health professional. Finally, at the participant’s request, the research team may provide oral, personalised feedback (general data and information regarding their participation).

#### Dissemination

We will follow open science transparency guidelines. Thus, we plan to preregister the main analyses before the end of data collection or, at the very least, before conducting the analyses. The results of the present study will be published in open-access, peer-reviewed academic journals. We will also organise symposia and poster presentations at key conferences in the field of child and adolescent mental health, as well as at meetings focusing on EMA. Additionally, findings will be disseminated to practitioners through the institutes’ close ties with other clinics and the educational sector. We further intend to make the main findings accessible to the general public in online and print magazines, at public events and on social media. A youth council will be involved in preparing the content and selecting the most effective media or way to reach the widest possible audience.

#### Scientific relevance

By using multimodal naturalistic methods with high ecological validity, the proposed project has the potential to add to the small evidence base of the distinction between tonic and phasic irritability in developmental psychopathology. In addition, the EMA allows for the investigation of within- and between-person variability in tonic and phasic irritability, and to explore their temporal sequences and direct precursors of the components of irritability as well as specify their natural context of expression. Moreover, using new multimodal technology to clinically characterise tonic and phasic irritability might inform the question whether tonic and phasic irritability are part of the same continuum or whether they represent two separate phenomena.^34^ Thus, this study will yield crucial data to develop new measures^13^ of tonic and phasic irritability.

In line with the most recent view of psychiatric disorders as continuous and dimensional phenomena rather than clearly delineated categorical entities, the proposed project thus takes a dimensional approach to quantification and severity of psychiatric symptomatology, abandoning the purely categorical diagnostic approach. Moreover, the naturalistic approach, including the parental perspective as well as using passive smartphone sensing, our project will lead to a better understanding of the contextual and interpersonal factors associated with irritability. Most importantly, our study is more likely to give insights into the psychophysiology of psychiatric disorders than approaches which examine disorders in a categorical manner.^123 124^ The proposed project, therefore, leverages the full spectrum of irritability in the help-seeking clinical sample and healthy control sample in combination with state-of-the-art clinical, cognitive, psychophysiological and neurobiological assessments, to contribute to the limited understanding of the underlying mechanisms of tonic and phasic irritability. Clinical re-evaluation of study participants will further allow us to determine the predictive value of tonic and phasic irritability for current and future psychopathology. The proposed project might further constitute the basis of a future cohort study. There is great potential in following these children up into adolescence and adulthood to understand the developmental pathways related to tonic and phasic irritability and its later consequences. A cohort study would further help to identify the best targets for early personalised intervention, reducing the negative long-term outcomes.

#### Broader impact of the study

One or two in ten children worldwide experience mental disorders.^125^,^127^ About 50% of mental disorders begin before adolescence and neuropsychiatric conditions are the leading cause of disability in youth.^126^ The prevalence of irritability has been reported to be rising substantially in community samples.^5^ Hence, obtaining a fine-grained understanding of the psychopathology of tonic and phasic irritability and pathophysiological mechanisms of one of the most common childhood psychiatric symptoms lays the foundation for intervening early. As such, childhood represents an early window of opportunity for the prevention of negative long-term outcomes for the affected individuals.^19^ For instance, specific cognitive remediation targeting specific self-control deficits,^128 129^ biofeedback^130 131^ or virtual reality techniques^132^ targeting specific psychophysiological markers of tonic and phasic irritability could be interesting future approaches based on the proposed project. Likewise, ecological momentary interventions or just-in-time adapted interventions^133^ could be developed based on the acquired knowledge from this study.
