# Supplementary material for: Understanding tonic and phasic irritability in developmental psychopathology among help-seeking children and adolescents in Switzerland: Protocol for the longitudinal multimodal UTOPICA study
Source: BMJ Open. 2026 Apr 22;16(4):e111168. doi: 10.1136/bmjopen-2025-111168 (PMC13110582; doi:10.1136/bmjopen-2025-111168)
Supplement: Supplementary file 1 [file bmjopen-2025-111168.reviewer_comments.pdf]

PEER REVIEW HISTORY

BMJ Open publishes all reviews undertaken for accepted manuscripts. Reviewers are asked to complete a checklist review form and are provided with free text boxes to elaborate on their assessment. These free text comments are reproduced below.

ARTICLE DETAILS

Title (Provisional)

Understanding tonic and phasic irritability in developmental psychopathology among help-seeking children and adolescents in Switzerland: protocol for the longitudinal multimodal UTOPICA study

Authors

Urben, Sebastien; Giovannini, Julia; Dietsche, Aline; Abi-Kheir, Marion; Schmidt, Leon; Koenig, Julian; Stringaris, Argyris; Plessen, Kerstin Jessica; Kaess, Michael; Mürner- Lavanchy, Ines

---

VERSION 1 - REVIEW

**Reviewer**	**1**
**Name**	**Shin, Mirim**
**Affiliation**	**The University of Sydney**
**Date**	**19-Jan-2026**
**COI**	**None**

This protocol describes an ambitious study examining tonic and phasic irritability in children aged 8-14 years using multimodal assessments, including clinical interviews, questionnaires, cognitive tasks, autonomic measures, and ambulatory assessment over 14 days with a one-year follow-up. The research addresses an important gap in developmental psychopathology, and the integration of within-person and between-person approaches is a notable strength. Given the data collection has begun, my comments focus primarily on clarifications rather than requests for methodological changes.

Abstract

• The abstract states “multilevel regression, as well as linear mixed modelling” as if these are distinct approaches, but linear mixed models are a form of multilevel regression.

Introduction

• The introduction spans approximately 7 pages and contains substantial repetition that should be addressed. Requires significant reduction.

• Section 1.7 presents three aims but does not state specific, testable hypotheses.

• The broader goal of developing “early identification and intervention” (Section 1.6) implies causal assumptions that are not explicitly stated. Do authors test causal hypotheses or establish predictive associations?

• The abbreviation AA (Ambulatory Assessment) is defined and used appropriately, though “Ecological Momentary Assessment (EMA)” may be more familiar to some readers.

Methods

• The protocol states n=40 healthy children will be recruited, but the operational definition of “healthy” is not provided.

• There is no mention of whether healthy controls will be comparable age/sex distributions to the clinical sample.

• Table 1 lists “drug naïve” as an inclusion criterion, but no rationale is provided.

• There is approximately 50 weeks between the end of the ambulatory assessment phase (Week +2) and the one-year follow-up (Week +52) with no active data collection. Section 2.3.5 indicates that therapy receipt and “significant life events” will be queried retrospectively at follow-up, but this approach is subject to recall bias.

• The procedure states that financial compensation will be provided “in form of vouchers to avoid direct monetary compensation that might be used inappropriately”. Please clarify what “inappropriate use” is being avoided.

• The TAPIS will be used to distinguish phasic and tonic irritability, but no details about its subscales or scoring are provided.

• For ESM measures, Section 2.3.4 mentions items measuring “affect (current and since the last prompt)” and “aggression”, but it is unclear which specific items capture tonic versus phasic irritability

• It seems that there are some discrepancies between Table 2 and the Methods text. Also, Table 2 should have clearer notation (e.g., text states sleep is measured during ESM but Table 2 shows sleep only at baseline; what is “<0”?)

• In in-situ assessment, what is the rationale for the 60%/40% rigged/accurate feedback ratio?

• For children using parents’ smartphones for ESM, how is data privacy maintained? Can parents view their children’s responses?

• In power analysis, please provide source of pilot estimates.

• There is no description of how compliance with the ambulatory assessment will be monitored or promoted.

• While Section 2.4.5 mentions assumed compliance and dropout rates, there is no explicit missing data handling plan.

• Is the study preregistered?

• Justify why one year is adequate for the stated aim of examining “future psychopathology”.

• The description of PPI in dissemination is vague.

• The figure is conceptually clear but would benefit from further improvements to its presentation.

• Given that irritability is associated with suicidality, please clarify how will participants who develop significant suicidal ideation during the ambulatory phase be identified and managed?

• Given the two-centre design with different languages, please address how will measurement equivalence across languages be established or assessed?

VERSION 1 - AUTHOR RESPONSE

Reviewer 1: Dr. Mirim Shin, The University of Sydney

This protocol describes an ambitious study examining tonic and phasic irritability in children aged 8-14 years using multimodal assessments, including clinical interviews, questionnaires, cognitive tasks, autonomic measures, and ambulatory assessment over 14 days with a one-year follow-up. The research addresses an important gap in developmental psychopathology, and the integration of within-person and between-person approaches is a notable strength. Given the data collection has begun, my comments focus primarily on clarifications rather than requests for methodological changes.

Authors’ response: Thank you for the encouraging feedback and the relevant comments that help us to clarify the description of our study protocol.

Abstract

• The abstract states “multilevel regression, as well as linear mixed modelling” as if these are distinct approaches, but linear mixed models are a form of multilevel regression.

Authors’ response: Thank you for this helpful suggestion. We changed the sentence to contain the appropriate description. Now it reads:

> *“Statistical analyses will include multilevel regression (e.g., linear mixed modelling).”*

Introduction

• The introduction spans approximately 7 pages and contains substantial repetition that should be addressed. Requires significant reduction.

Authors’ response: Accordingly, we reworked the introduction in order to avoid repetition and reduce its length.

• Section 1.7 presents three aims but does not state specific, testable hypotheses.

Authors' response: Accordingly, we added testable hypotheses in the section 1.8. Now it reads (pp. 11-12):

> *"1.8 The current research project*
>
> *The overall objective of this research project is to develop a fine-grained, ecologically-valid, and multimodal characterization of tonic and phasic irritability in order to better understand the differential role of the two components of irritability in developmental psychopathology. In particular, through ESM assessment, our* ***first aim*** *is to validate the tonic - phasic distinction of irritability at the within-person level and to determine the temporal dynamics as well as the natural context of expression of the two components. We hypothesize that the tonic and phasic components of irritability measured with ESM will be strongly and consistently associated with their respective well-established clinical measures, thereby providing new reliable and complementary measures. We further assume that high levels of tonic irritability will precede phasic irritability (i.e., temper tantrum). Moreover, contextual information (including parenting) about the current psychosocial environment might be differently related to tonic versus phasic irritability.*
>
> *Our* ***second aim*** *is to examine whether distinct cognitive and autonomic correlates of tonic and phasic irritability exist, both at the within-person level and at the between-person level. Related to this aim, we hypothesize that experimental measures of ambulatory cognitive control will be more strongly associated with phasic than tonic irritability. From the "in-situ" assessment, we expect that HRV reactivity will be more specifically related to phasic aspects of irritability (clinical and ESM assessments), whereas HRV recovery (more related to emotion regulation) will be associated with tonic irritability (ESM and well-established clinical assessment). Finally, by means of the autonomic EMA, we expect to identify autonomic dysregulation concurrent with tonic irritability but probably preceding episodes of phasic irritability.*
>
> *Finally, extending previous studies examining the distinction between tonic and phasic irritability in clinical samples, using our ecologically-valid multimodal approach, our* ***third aim*** *is to use the fine-grained understanding of irritability to examine the unique and different associations of its tonic and phasic facets with current and future psychopathology, both at the within- and between-person level. We hypothesize that tonic irritability will be independently related to*

current and future internalizing psychopathology, including depressive, anxiety symptoms, and suicidal ideation, while phasic irritability can be independently associated with current and future externalizing symptoms, hyperactivity symptoms, oppositional, conduct problems, and impulsive suicide attempts. We will further explore whether EMA measures and "in-situ" assessment are more strongly associated with current and future psychopathologies compared to well-established clinical assessments. Finally, we expect more stability in the phasic compared to the tonic component of irritability over the long-term."

• The broader goal of developing "early identification and intervention" (Section 1.6) implies causal assumptions that are not explicitly stated. Do authors test causal hypotheses or establish predictive associations?

Authors' response: Regarding the micro- and macro-longitudinal design we will be able to establish predictive associations, thus, allowing for developing early identification and intervention. This is more explicitly described in the revised form of the manuscript. Notice that we reduce also drastically this section. Now it reads (p. 10):

"A central question is whether tonic and phasic irritability reflect different intensity levels along a single continuum or constitute related but distinct phenotypes[34]. This study uses ecologically valid, multimodal, and multi-informant assessments, as well as EMA, to characterize both dimensions. By integrating cognitive, psychosocial, psychophysiological, and autonomic measures, the study aims to clarify within-person dynamics and identify proximal markers of tonic and phasic irritability. This approach will also allow for the examination of their concurrent and longitudinal associations with clinical outcomes, with the goal of laying a foundation for future work improving early identification and intervention targets."

• The abbreviation AA (Ambulatory Assessment) is defined and used appropriately, though "Ecological Momentary Assessment (EMA)" may be more familiar to some readers.

Authors' response: Accordingly, we replaced ambulatory assessment by EMA as indeed, it refers to a more familiar term in the international literature.

Methods

• The protocol states n=40 healthy children will be recruited, but the operational definition of "healthy" is not provided.

Authors' response: Healthy controls refer to children (or families) not seeking help or support and being free from psychiatric disorder as assessed by the K-SADS. This information has been updated in the revised form of the manuscript, p. 12:

“Moreover, we will recruit n = 40 healthy children (i.e., not seeking help and no diagnosed psychiatric disorder).”

• There is no mention of whether healthy controls will be comparable age/sex distributions to the clinical sample.

Authors’ response: We intend to analyze the data using a dimensional framework of psychopathology. By recruiting participants spanning a broad range of irritability severity, our goal is not to compare predefined groups, but rather to examine continuous associations between irritability and other psychopathological symptoms and understanding the cognitive underpinning of irritability. It is more explicitly stated in the section “*1.8 The current research project*”.

• Table 1 lists “drug naïve” as an inclusion criterion, but no rationale is provided.

Authors’ response: We aim to characterize the associations between these phenomena without the confounding influence of medication that may alter emotional and behavioral functioning, as well as psychophysiology. We added this information as a footnote of the Table 1.

• There is approximately 50 weeks between the end of the ambulatory assessment phase (Week +2) and the one-year follow-up (Week +52) with no active data collection. Section 2.3.5 indicates that therapy receipt and “significant life events” will be queried retrospectively at follow-up, but this approach is subject to recall bias.

Authors’ response: Indeed, while parents may not recall every detail from the past year, it is reasonable to assume that they remember major life events and therapeutic interventions provided to their child. Therefore, substantial recall bias is unlikely to meaningfully compromise data reliability.

• The procedure states that financial compensation will be provided “in form of vouchers to avoid direct monetary compensation that might be used inappropriately”. Please clarify what “inappropriate use” is being avoided.

Authors’ response: For ethical reasons, we provided vouchers rather than direct financial compensation to prevent the remuneration from being used to purchase cigarettes, alcohol, or other illegal substances. This information has been added, p.13.

• The TAPIS will be used to distinguish phasic and tonic irritability, but no details about its subscales or scoring are provided.

Authors’ response: This has been clarified in the revised version of the manuscript, p. 15.

• For ESM measures, Section 2.3.4 mentions items measuring “affect (current and since the

last prompt)" and "aggression", but it is unclear which specific items capture tonic versus phasic irritability

Authors' response: Accordingly, we added information on which items capture tonic versus phasic irritability.

• In in-situ assessment, what is the rationale for the 60%/40% rigged/accurate feedback ratio?

Authors' response: First, this ratio adheres to standardized procedures described in the literature to ensure methodological rigor and to facilitate comparability with previous research. Moreover, a 60% rate of non-contingent negative feedback reliably induces frustration while avoiding excessive distress. This proportion has been shown to degrade performance and modulate neural markers of reward and frustrative non-reward, particularly in vulnerable individuals. The 60/40 non-reward/reward balance delivers a strong frustrative signal while preserving enough accurate positive feedback to maintain task credibility and participant engagement. See changes at p. 17.

• For children using parents' smartphones for ESM, how is data privacy maintained? Can parents view their children's responses?

Authors' response: Children complete the questionnaires directly within the REDCap® environment. Once their responses are entered and submitted, parents cannot access or view their child's answers.

• In power analysis, please provide source of pilot estimates.

Authors' response: The pilot study consists of our own unpublished data, which provided us with a realistic estimate of statistical power. This information has been added to the manuscript, p. 22.

• There is no description of how compliance with the ambulatory assessment will be monitored or promoted.

Authors' response: We agree that ensuring compliance is essential and we implemented several procedures. Daily monitoring of REDCap® entries will be performed to verify that participants complete the scheduled questionnaires. Additionally, every two to three days, we will also monitor synchronization status for both mobile applications (i.e., Labfront, Garmin Connect) involved in the study. When a decrease in compliance will be detected, participants will receive an SMS reminder. However, if compliance does not improve, the research team will call the participant directly to identify potential difficulties, provide assistance, and encourage re-engagement during the ambulatory phase. Now it reads (p. 19).

"To maximize EMA response compliance, we will monitor daily REDCap entries. Additionally, every two or three days, we will review the synchronization status for both mobile applications (i.e., Labfront, Garmin Connect). Participants will receive an SMS reminder on their smartphone if a decline in compliance is detected. If compliance does not improve, a member of the research team will contact the participant by phone to identify potential difficulties, offer support, and encourage re-engagement during this phase."

• While Section 2.4.5 mentions assumed compliance and dropout rates, there is no explicit missing data handling plan.

Authors' response: We added more information about the handling of missing data in a new section, 2.4.6 (p. 23):

"Missing data will be minimized through electronic data capture (via REDCap® with built-in range checks and completion indicators). Patterns of missingness will be assessed. If data are missing at random, multiple imputation will be applied. If data are missing completely at random (MCAR), analyses may additionally be performed using expectation-maximation procedures. Results from analyses on the imputed dataset, will be compared with complete-case analyses as part of a sensitivity analysis."

• Is the study preregistered?

Authors' response: No, the study is not yet preregistered. We see this manuscript as a form of study registration. Moreover, each publication resulting from this data collection will be preregistered, in line with best practices. We will adhere as closely as possible to open science principles.

• Justify why one year is adequate for the stated aim of examining "future psychopathology".

Authors' response: In children and young adolescents, substantial changes in symptom severity and clinically relevant onsets typically occur within a 12-month window, and prior longitudinal cohorts have successfully used 1-year follow-ups to examine "*emerging*" or "*future*" psychopathology in this age group. Given the intensive, multimodal nature of our protocol, a one-year interval offers the optimal balance between capturing meaningful developmental changes in irritability-related psychopathology and maintaining feasibility, retention, and ethical acceptability, consistent with current youth mental-health and early-risk-prediction studies. We added this information in the revised version of the manuscript (p. 13). That said, a longer follow-up period would even enhance the conclusions on developmental psychopathology drawn from this study.

• The description of PPI in dissemination is vague.

Authors' response: Accordingly, we added more information about PPI in the dissemination phase. Now it reads (p. 25):

> *"A youth council will be involved in the preparation of the content and the best media or way to diffuse the main findings in the largest audience possible."*

• The figure is conceptually clear but would benefit from further improvements to its presentation.

Authors' response: Accordingly, we rework the figure.

• Given that irritability is associated with suicidality, please clarify how will participants who develop significant suicidal ideation during the ambulatory phase be identified and managed?

Authors' response: No close clinical monitoring is conducted as part of the ambulatory assessment phase. A mental health professional evaluates each participant at baseline, and any clinically significant findings are addressed in accordance with standard care procedures. If an acute crisis is identified, the participant is immediately referred for appropriate clinical intervention. Further, such situations constitute an exclusion criterion.

• Given the two-centre design with different languages, please address how will measurement equivalence across languages be established or assessed?

Authors' response: We apply a standardized protocol to ensure consistency in data collection across both sites. All instruments used in the study are well-validated and widely employed in the literature and we will use harmonized French and German versions (including identical administration procedures). We will explicitly assess measurement equivalence across languages/sites prior to primary analyses. Specifically, we will test multi-group measurement invariance (French vs German) using confirmatory factor analysis (CFA) for multi-item scales, examining sequentially configural, metric, and scalar invariance. If full invariance is not supported, we will examine sources of misfit and apply partial invariance (freeing non-invariant loadings/intercepts/thresholds) as needed. Main analyses will then be conducted using invariant (or partially invariant) measurement models; as a robustness check, we will additionally run models adjusting for site/language (and, where relevant, site/language × predictor interactions) to ensure conclusions are not driven by language-related measurement differences. We added this information in the revised form of the manuscript.

Thus, in procedure we wrote (p. 13):

> *"A standardized data-collection protocol was implemented to ensure methodological consistency across sites. All assessment instruments*

> *have demonstrated strong psychometric properties and are validated in prior research."*

As well in the data analyses section, we added (p. 19):

> *"We will assess cross-language measurement invariance (French vs German) for multi-item scales using multi-group CFA, testing configural, metric, and scalar (threshold) invariance based on model fit and ΔCFI/ΔRMSEA. If inter-site differences are detected, site will be included as a covariate in the statistical models to control for potential confounding."*
